# Chronic Ethanol Consumption Inhibits Glucokinase Transcriptional Activity by *Atf3* and Triggers Metabolic Syndrome in Vivo*

**DOI:** 10.1074/jbc.M114.585653

**Published:** 2014-07-29

**Authors:** Ji Yeon Kim, Joo-Yeon Hwang, Dae Yeon Lee, Eun Hyun Song, Keon Jae Park, Gyu Hee Kim, Eun Ae Jeong, Yoo Jeong Lee, Min Jin Go, Dae Jin Kim, Seong Su Lee, Bong-Jo Kim, Jihyun Song, Gu Seob Roh, Bin Gao, Won-Ho Kim

**Affiliations:** From the ‡ Division of Metabolic Disease, Center for Biomedical Science,; § Division of Structural and Functional Genomics, Center for Genomic Science, National Institutes of Health, Osong-eup, Cheongwon-gun, Chungbuk 363-951, Korea,; the Departments of ** Psychiatry and; ‡‡ Endocrinology, College of Medicine, Catholic University, Bucheon 420-743, Korea,; the §§ Department of Anatomy and Neurobiology, Institute of Health Sciences, Gyeongsang National University, Jinju, Gyeongnam 660-751, Korea,; the ¶ Department of Biotechnology, College of Life Sciences and Biotechnology, Korea University, Seoul 136-701, Korea,; the ‖ Division of Cardiology, Department of Internal Medicine, Chungbuk National University School of Medicine, Cheongju 361-763, Korea, and; the ¶¶ Laboratory of Liver Diseases, National Institute on Alcohol Abuse and Alcoholism, Bethesda, Maryland 20892

**Keywords:** Gene Expression, Gene Therapy, Glucokinase, Metabolic Disease, Pancreatic Islet, Type 2 Diabetes

## Abstract

Chronic ethanol consumption induces pancreatic β-cell dysfunction through glucokinase (Gck) nitration and down-regulation, leading to impaired glucose tolerance and insulin resistance, but the underlying mechanism remains largely unknown. Here, we demonstrate that *Gck* gene expression and promoter activity in pancreatic β-cells were suppressed by chronic ethanol exposure *in vivo* and *in vitro*, whereas expression of activating transcription factor 3 (Atf3) and its binding to the putative Atf/Creb site (from −287 to −158 bp) on the *Gck* promoter were up-regulated. Furthermore, *in vitro* ethanol-induced Atf3 inhibited the positive effect of Pdx-1 on *Gck* transcriptional regulation, enhanced recruitment of Hdac1/2 and histone H3 deacetylation, and subsequently augmented the interaction of Hdac1/Pdx-1 on the *Gck* promoter, which were diminished by *Atf3* siRNA. *In vivo Atf3*-silencing reversed ethanol-mediated *Gck* down-regulation and β-cell dysfunction, followed by the amelioration of impaired glucose tolerance and insulin resistance. Together, we identified that ethanol-induced *Atf3* fosters β-cell dysfunction via *Gck* down-regulation and that its loss ameliorates metabolic syndrome and could be a potential therapeutic target in treating type 2 diabetes. The *Atf3* gene is associated with the induction of type 2 diabetes and alcohol consumption-induced metabolic impairment and thus may be the major negative regulator for glucose homeostasis.

## Teaser

**Background:** Chronic ethanol consumption induces pancreatic β-cell dysfunction and metabolic syndrome.

**Results:** Ethanol-induced Atf3 inhibits glucokinase transcriptional activity through direct binding or Atf3/Pdx-1/Hdac1 axis on glucokinase promoter.

**Conclusion:** ATf3 fosters β-cell dysfunction via *Gck* down-regulation and triggers T2D, which is ameliorated by *in vivo Atf3* silencing.

**Significance:** The presented data uncover a new role for Atf3 as a potential therapeutic target in treating type 2 diabetes.

## Introduction

Chronic ethanol consumption is well established as an independent risk factor for type 2 diabetes (T2D)^2^ and has been suggested to cause increased adiposity, impaired glucose homeostasis, and insulin resistance (1, 2). Several studies have demonstrated that light-to-moderate ethanol consumption is associated with a lower risk of T2D through increased glucose-stimulated insulin secretion and insulin sensitivity in peripheral tissues (3, 4). However, it is generally believed that the administration of moderate amounts of ethanol to rodents with metabolic disorders may increase the risk of complications in metabolic diseases or diabetes (5, 6). Based on several previous studies, pancreatic β-cells may be sensitive to ethanol-induced oxidative stress associated with the production of reactive oxygen species, which is one of the earliest events in glucose intolerance and is associated with mitochondrial dysfunction (7, 8). However, the mechanism by which chronic ethanol consumption induces pancreatic β-cell dysfunction and how such induced β-cell dysfunction contributes to the progression of T2D remain largely unknown.

Glucokinase plays a critical role in blood glucose homeostasis, serving as a glucose sensor for glucose-stimulated insulin secretion in pancreatic β-cells and as the major regulator of glucose uptake in hepatocytes (9, 10). Recently, we suggested that ethanol consumption may induce pancreatic β-cell dysfunction and apoptosis through Gck nitration and down-regulation, correlated with impaired glucose responsiveness and insulin resistance (11). Although we demonstrated that ethanol-induced Atf3 in pancreatic β-cells may function as an upstream regulator of *Gck* down-regulation, little is known about the exact role and regulatory mechanism of Atf3 in ethanol-induced *Gck* down-regulation.

Atf3, a member of the Atf/Creb family of transcription factors, regulates gene expression by binding to the consensus Atf/Creb cis-regulatory element via a basic leucine zipper domain (12). Given its frequent induction by various cellular stressors, ectopic expression of Atf3 in heart, liver, and pancreatic β-cells causes cardiac enlargement, liver or pancreatic β-cell dysfunction and apoptosis, impaired glucose metabolism, and diabetes (13). Although pancreatic duodenal homeobox-1 (Pdx-1) and sterol regulatory element-binding protein-1c directly bind to specific elements of the pancreatic and liver *Gck* promoter, respectively, and are positive regulators for *Gck* gene expression (14, 15), the relevant upstream activator or repressor regulators involved in *Gck* transcriptional regulation are little known. We previously suggested that lipotoxicity-induced Atf3 may be associated with the inhibition of Pdx-1-mediated transcriptional activity (16), but the precise action mechanisms of Atf3 are still not clear. Generally, transcription is regulated by various complex processes that require cooperation between transcription factors and co-activators or co-repressors that modulate histone structure (17). Histone modification via acetylation, phosphorylation, and methylation has been implicated in increased or decreased accessibility to transcription machinery, thereby leading to the repression or activation of gene expression (18). The β-cell-specific transcription factor Pdx-1 has been shown to interact with the histone acetyltransferase p300/Cbp, and this interaction has been demonstrated to be important for *Gck*-mediated insulin and Glut2 expression (19). We also demonstrated that endoplasmic reticulum stress-induced Atf3 inhibits Pdx-1-mediated PBE/Luc activity or *Gck*/Luc activity by inhibiting the interaction between Pdx-1 and p300, which leads to β-cell dysfunction (20). These results prompted us to investigate whether ethanol-induced Atf3 may also act as a transcriptional repressor of *Gck* gene expression via histone modification, leading to pancreatic β-cell dysfunction and apoptosis. This study provides molecular insight into the mechanism by which chronic ethanol-induced Atf3 inhibits the transcriptional activity of *Gck* through direct binding to the consensus Atf/Creb-binding site and the formation of an Atf3/Pdx-1/Hdac1/2 axis at the *Gck* promoter with the deacetylation of histone H3. Clear evidence for the amelioration of these events by *Atf3* silencing using *in vivo*-jetPEI siRNA delivery system may offer a therapeutic strategy to combat the impaired glucose metabolism, insulin insensitivity, and hyperglycemia associated with chronic ethanol consumption.

## EXPERIMENTAL PROCEDURES

### 

#### 

##### Cell Lines and Isolated Islet Cells

Rat INS-1 pancreatic β-cells were cultured in RPMI 1640 medium containing 11.1 mm glucose supplemented with 10% fetal bovine serum, 2 mm glutamine, 1 mm sodium pyruvate, 55 μm β-mercaptoethanol, 10 mm HEPES, 100 IU/ml penicillin, and 100 μg/ml streptomycin (Invitrogen). All experiments were performed between passage P4 and P20. Islet cells were isolated from overnight-fasted C57BL/6 mice using a previously described collagenase digestion technique (11). All antibodies were obtained from Cell Signaling Technology (Beverly, MA) or Santa Cruz Biotechnology Inc. (Santa Cruz, CA), and chemicals were purchased from Sigma.

##### In Vitro Treatment of Ethanol

To determine the concentrations and time points of ethanol showing positive effects on cell physiology (insulin content and ATP production) and the changes of phenotypes (apoptosis), we have treated the INS-1 cells or isolated islet cells with ethanol at the different concentrations (0, 25, 50, 100, and 200 mm) and time points (0, 6, 12, 24, and 48 h). Thereafter, the effective concentration (100 mm) and time (24 h) of ethanol, which show the most effective effects on the decrease of insulin content and ATP production and the increase of apoptosis, were selected. The treatment of ethanol with less than 50 mm or more than 100 mm did not have effective effects. Although there are still conflicting claims in the relative calculation between millimolar and *in vivo* percentage (%) of blood alcohol levels (22), several previous studies have shown that the selected 100 mm ethanol actually corresponds to about 0.46% (23), which can yield signs of intoxication in *in vivo* organs. The selected concentration (100 mm) and time (24 h) of ethanol is currently accepted and considered as an acute ethanol consumption in an *in vitro* model (24, 25). When cells were treated with 100 mm ethanol, the final media contained the volume of treated ethanol. However, when cells were treated with ethanol, alcohol exposure of cells may be hampered by evaporation of the alcohol. The fluctuation of alcohol concentration and ethanol effects on the cells was due to evaporation. To avoid this, investigators used settings where ethanol was added into the culture media and the cell culture plates were maintained for the entire duration of stimulation in a microclimate chamber at 37 °C with a gas mixture and an alcohol atmosphere (26).

##### Animals

C57BL/6J male mice (6 weeks old) originally purchased from The Jackson Laboratory (Bar Harbor, ME) were used in all experiments. Individually caged mice were placed on a Lieber-Decarli regular liquid diet (Dyets; control diet number 710027 or ethanol diet number 710260). Mice were pair-fed with the control *versus* 5% (v/v) ethanol diet for 8 weeks as reported previously (11). All animal experiments were conducted in accordance with guidelines from the Korean National Institute of Health Animal Facility.

##### Plasmids

Human wild-type *ATF3* and *ATF3*(ΔC,101–181) with a C-terminal deletion and cDNA expression vectors were generous gifts from Dr. T. Hai (Ohio State University). *ATF3*(ΔN, 1–100) cDNA was amplified by PCR using the primer forward 5′-GGAATTCGCAAAGTGCCGAAACAAG-3′ and reverse 5′-ACGCGTCGACTTAGCTCTGCAATGTTCC-3′ and cloned into pcDNA3 or pEGFP-C2 (Invitrogen). *Pdx-1* expression vector (pcDNA3/*Pdx-1*) was provided by Dr. T. Stein (Ohio State University). Rat *Gck* promoter reporter pRGP-1003/Luc, pRGP-404/Luc, pRGP-287/Luc, and pRGP-84/Luc were kindly provided by Dr. Y. Ahn (Yonsei University College of Medicine). pRGP-158/Luc vector, deletion mutant, or site-mutated plasmids were constructed in the pGL3-*Gck*/Luc plasmid by the two-step PCR method or QuikChange site-directed mutagenesis (Stratagene) according to the manufacturer's instructions. The used primer sets for each mutated construct are follows: pRGP-158/Luc, forward 5′-TTTCTCTATCGATAGGTACCGGTGACAGGAGTAGAGAG-3′ and reverse 5′-CCAAGCTGATCTCGAGCCCG-3′; *mCREB1,* forward 5′-CTGCTCCTTAGTAAGTGATACAGGCACTAAGGCAC-3′ and reverse 5′-GTGCCTTAGTGCCTGTATCACTTACTAAGGAGCAG-3′; *mCREB2*, forward 5′-GTAACAGGCACTAAGGCACAAACCTGGGAACTGAGCAG-3′ and reverse 5′-CTGCTCAGTTCCCAGGTTTGTGCCTTAGTGCCTGTTAC-3′; *mCREB3*, forward 5′-CACTGACCTGGGAACTGGCCAGGTGGTAATGTCTAC-3′ and reverse 5′-GTAGACATTACCACCTGGCCAGTTCCCAGGTCAGTG-3′; *mCREB4*, forward 5′-CTGGCAGTCACTGCAGATACAGGGTGACAGAGTG-3′ and reverse 5′-CACTCTGTCACCCTGTATCTGCAGTGACTGCCAG-3′; *mCREB5*, forward 5′-CACTGCAGTGACAGGGATACAGAGTGGTCCCATG-3′ and reverse 5′-CATGGTGACCACTCTGTATCCCTGTCACTGCAGTG-3′; *mCREB6*, forward 5′-CACTGCAGATACAGGGATACAGAGTGGTCACCAT-3′ and reverse 5′-ATGGTGACCACTCTGTATCCCTGTATCTGCAGTG-3′; *mAP1,* forward 5′-GTCTACCAGGCTGGCATACACTGCAGTGACAGGG-3′ and reverse 5′-CCCTGTCACTGCAGTGTATGCCAGCCTGGTAGAC-3′; *mAP2,* forward 5′-GACAGGGTGACAG AGTGTACACCATGGTGACAGGAG-3′ and reverse 5′-CTCCTGTCACCATGGTGTACACTCTGTCACCCTGTC-3′; *mPdx-1*, forward 5′-CTGCATGGTGGCTCTAGATATAGAATGGTCACCATAG-3′ and reverse 5′-CTATGGTGACCATTCTATATCTAGAGCCACCATGCAG-3′; Δ*Pdx-1,* forward 5′-GTTTTCTGCATGGTGGAATGGTCACCATAGAAAC-3′ and reverse 5′-GTTTCTATGGTGACCATTCCACCATGCAGAAAAC-3′; and Δ*ATF*, forward 5′-GGCTGGCAGTCACTGCAGTGGTCACCATGGTG-3′ and reverse 5′-CACCATGGTGACCACTGCAGTGACTGCCAGCC-3′.

##### Luciferase Activity Analysis

After transfection or treatment, the cells were lysed, and luciferase activity was measured using the Luciferase Assay System (Promega, Madison, WI).

##### RNA Interference and Transient Transfection

The human (sc-29757) or rat *Atf3*- (sc-72029) and mouse (sc-38761) or rat *Pdx-1* (sc-10840) siRNAs were purchased from Santa Cruz Biotechnology. The cells were plated at 60–70% confluence and transfected with siRNA complexes or only with transfection reagents using Lipofectamine 2000 (Invitrogen) following the protocol recommended by the manufacturer.

##### RT-PCR and Quantitative RT-PCR

Total RNA was extracted from INS-1 cells and islet cells using TRIzol reagent (Invitrogen) and was subjected to reverse transcription using reverse transcriptase (Promega) at 42 °C for 1 h, and the resulting cDNA was amplified by PCR using gene-specific primers. The primers were designed using the Primer 3 program and cross-checked by a BLAST search of the NCBI database. The primer sets are as follows: mouse *Gck* (NM_010292), forward 5′-GTGCTGCTCAAGCTGGTAGA-3′ and reverse 5′-GCGATTTATGACCCCCGCTA-3′; rat *Gck* (NM_001270849), forward 5′-GACAGTC CTCACCTGCAACA-3′ and reverse 5′-GCATTTGTTGGTGCCCAGAG-3′; mouse *Glut2* (NM_031197), forward 5′-ACCGGGATGATTGGCATGTT-3′ and reverse 5′-GAACACGTAAGGCCCAAGGA-3′; rat *Glut2* (NM_012879), forward 5′-ACTGGCACATCCTACTTGGC-3′ and reverse 5′-CAGTCGACGCCTCTTCCTTT-3′; mouse *Atf3* (NM_007498), forward 5′-GGAAAACTGGCTTCCTGTGC-3′ and reverse 5′-TGGCCATTGGACAACCTCAA-3′; rat *Atf3* (NM_012912), forward 5′-CTCTAGCCGCTCTCTGGACC-3′ and reverse 5′-CGGCATTCACACTCTCCAGT-3′; mouse *Gapdh* (NM_008084), forward 5′-GGTTGTCTCCTGCGACTTCA-3′ and reverse 5′-TAGGGCCTCTCTTGCTCAGT-3′; and rat *Gapdh* (NM_017008), forward 5′-GGACCAGGTTGT CTCCTGTG-3′ and reverse 5′-ATTCGAGAGAAGGGAGGGCT-3′. The quantitative reverse transcriptase-PCR analysis was performed by using SYBR Green PCR Supermix (Bio-Rad) to detect the real time quantitative PCR products of reverse-transcribed cDNA according to the manufacturer's instructions (24). The primers are as follows: rat *Gck,* forward 5′-TCTACTTCCCCAACGACCCC-3′ and reverse 5′-GTTCATGTGCCCGTTGTGAG-3′; and rat *Glut2*, forward 5′-GTCCCAGTTTTCTGCAT GGT-3′ and reverse 5′-GAGTAGATGCCTCCCGTCAG-3′.

##### Electrophoretic Mobility Shift Assay (EMSA)

DNA mobility shift assays were performed as described previously (27). In brief, EMSA was performed in 20-μl volumes with 20 mm Tris-HCl, pH 7.9, 1.5% glycerol, 50 μg/ml BSA, 1 mm DTT, 0.5 mm phenylmethylsulfonyl fluoride, 2 μg of poly(dI-dC), 1 ng of ^32^P-labeled probe (5′-TGCAGTGACAGGGTGACAGA-3′), and 10 μg of nuclear extract. Reactions were incubated at 25 °C for 20 min and subsequently analyzed by electrophoresis through nondenaturing stock polyacrylamide gels (6 or 10%) in 0.5× TBE buffer containing 44.5 mm Tris-HCl, pH 8.2, 44.5 mm boric acid, and 1 mm EDTA. After the gel was pre-run at 100 V for 2 h, electrophoresis was performed at 270 V for 2 h at 4 °C. The gels were exposed to x-ray films using two intensifying screens at −70 °C.

##### Biochemical Analysis

Blood was collected by cardiac puncture from mice that had fasted overnight. Sera and relevant peripheral tissues were stored at −20 °C and used subsequently to assess the levels of triglycerides. Triglycerides were measured by colorimetric assays. For glucose tolerance test and insulin tolerance test, the mice were fasted at 8:00 a.m. for 6 h and then injected intraperitoneally with 1g/kg glucose or 1.5 units/kg regular human insulin, respectively. Blood samples were collected from the tail vein at time 0 (before injection), 30, 60, 90, and 120 min after injection, and the blood glucose level was measured using a portable glucose meter (Glucocard II Arkray, Kyoto, Japan) (11). The levels of insulin, ATP, and nitrite were also determined as described previously (11). Intracellular ROS generation was measured by using dichlorodihydrofluorescein diacetate (Molecular Probes, Eugene, OR) (11).

##### Immunoblots and Coimmunoprecipitation

Cells were lysed in RIPA buffer at 4 °C, vortexed, and then centrifuged at 16,000 rpm for 10 min at 4 °C. The supernatant was mixed in Laemmli loading buffer, boiled for 4 min, and then subjected to SDS-PAGE. For endogenous complexes, mitochondrial lysates (1 mg) fractionated from the cells were immunoprecipitated with 2 μg of antibody and immunoblotted.

##### Terminal Deoxynucleotidyltransferase-mediated dUTP Nick-end Labeling (TUNEL)

Apoptotic cells were detected using Apop Tag, an *in situ* apoptosis detection kit (Oncor, Gaithersburg, MD) as described previously (11).

##### Histopathology

For immunohistochemistry analysis, after pancreatic tissues were fixed, deparaffinized, and washed, sections were treated with diluted blocking serum for 20 min. Slides were incubated overnight at 4 °C in a humidified chamber with specific antibodies diluted in blocking serum. For immunocytochemistry analysis, the INS-1 cells were grown on glass coverslips for 24 h in a 6-well plate. After treatment, the cells were washed with cold PBS and fixed in paraformaldehyde (4% in PBS) for 15 min. All procedures were performed as described previously (11).

##### Chromatin Immunoprecipitation (ChIP)

The ChIP assays were performed according to the manufacturer's protocol (Upstate Biotechnology, Lake Placid, NY). In brief, after treatment, cells were cross-linked with 1% formaldehyde in medium for 15 min at 37 °C and then washed with ice-cold PBS and resuspended in 200 μl of SDS sample buffer containing protease inhibitor mixture. The suspension was sonicated 10 times for 30 s with a 1-min cooling period on ice between times and pre-cleared with 20 μl of protein A-agarose beads blocked with sonicated salmon sperm DNA for 30 min at 4 °C. The beads were removed, and the chromatin solution was immunoprecipitated overnight with anti-Atf3, Pdx-1, Stat5, Ac-K18 histone H3 (1766-1, Epitomics), Ac-K8 histone H4 (1796–1, Epitomics), and polymerase II (2035-1, Epitomics) antibodies at 4 °C, followed by incubation with protein A-agarose beads for an additional hour at 4 °C. The immunocomplexes were eluted with 100 μl of elution buffer (1% SDS and 0.1 mol/liter NaHCO_3_), and formaldehyde cross-links were reversed by heating at 65 °C for 6 h. Proteinase K was added to the reaction mixtures and incubated at 45 °C for 1 h. DNAs of the immunoprecipitates and control input DNAs were purified using a QIAquick PCR purification kit (Qiagen Inc.) and then analyzed by regular PCR using rat *Gck* promoter-specific primers. Alternatively, quantitative PCR was performed using the same primers in the presence of SYBR Green PCR Supermix (Bio-Rad) to detect the real time quantitative PCR products of chromatin immunoprecipitation samples according to the manufacturer's instructions (20, 28). The primer sequences for ChIP and qChIP are as follows: rat *Gck* promoter (−283 to −135), forward 5′-CGTAGAGGGCTCTGCTCCTT-3′ and reverse 5′-CAAAGGCCTCTCTACTCCTGTC-3′; rat *Gck* promoter (−127 to −28), forward 5′-GTCCCAGTTTTCTGCATGGT-3′ and reverse 5′-GAGTAGATGCCTCCCGTCAG-3′; and qChIP analysis of rat *Gck* promoter (−125 to +15), forward 5′-CCCAGTTTTCTGCATGGTGG-3′ and reverse 5′-CGAGCTCAGTCACAGTCGAT-3′.

##### In Vivo Atf3 Gene Silencing

Chemically synthetic siRNA against *Atf3* was synthesized by Dharmacon RNA Technology. A commercially available cationic polymer transfection reagent (*in vivo*-jetPEI^TM^, PolyPlus-Transfection, Illkirch, France) was used to deliver siRNA via intravenous injection (29–31). Briefly, 150 μg of siRNA diluted in 200 μl of 5% glucose solution was mixed with *in vivo*-jetPEI solution including transfection reagent jetPEI. The mixture was incubated for 15 min at room temperature to allow the complexes to form. This mixture was then injected into the ethanol-fed mice for 5 weeks once to six times with a 3-day interval between injections and continuously exposed to ethanol by 8 weeks. The scrambled siRNA solution mixed with *in vivo*-jetPEI solution served as a control siRNA. Three-day interval injection of siRNA was found to be optimum to sustain the effects of siRNA via preliminary experiments. The *in vivo* delivery efficiency of siRNA was assessed by performing RT-PCR and Western blotting in the removed tissues, lung, liver or pancreas, and isolated islet cells. The four siRNA candidates for mouse-specific *Atf3* are as follows: *Atf3* mouse-736, forward 5′-GCGGCGAGAAAGAAAUAATT-3′ and reverse 5′-UAAAGAGGUUCCUCUCGUCTT-3′; *Atf3* mouse-587, forward 5′-GCAGAAAGAGUCAGAGAAATT-3′ and reverse 5′-UUUCUCUGACUCUUUCUGCTT-3′; *Atf3* mouse-516, forward 5′-GCGGCGAGAAAGA AAUAAATT-3′ and reverse 5′-UUUAUUUCUUUCUCGCCGCTT-3′; and *Atf3* mouse-356, forward 5′-CACCCUUUGUCAAGGAAGATT-3′ and reverse 5′-UCUUCCUUGACAAAGGGUGTT-3′.

##### Statistical Analysis

For comparing values obtained in three or more groups, one-factor analysis of variance was used, followed by Turkey's post hoc test, and *p* < 0.05 was taken to imply statistical significance.

## RESULTS

### 

#### 

##### Chronic Ethanol Consumption Represses Gck Transcriptional Activity in Pancreatic β-Cells

**FIGURE 1.**
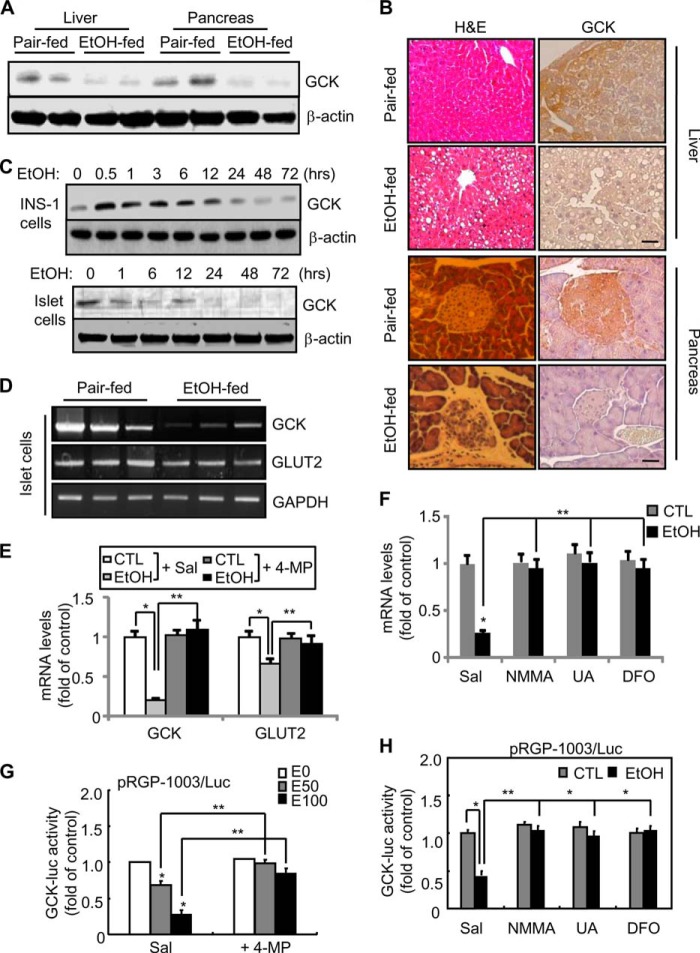
**Down-regulation of *Gck* expression in pancreatic β-cells of ethanol-fed mice.** 6-Week-old male C57BL/6J mice (*n* = 8, each group) were fed an ethanol diet for 8 weeks. *A,* liver and pancreas tissues of ethanol-fed mice were subjected to Western blotting for anti-Gck antibody. *B,* H&E staining (*left, scale bars,* 50 μm (liver) and 100 μm (pancreas)) and immunohistochemistry analysis for Gck (*right, scale bars,* 100 μm). *C,* INS-1 (*upper panel*) and isolated islet cells (*lower panel*) were treated with ethanol for the indicated time periods and subjected to Western blotting. *D,* RT-PCR in islet cells isolated from ethanol-fed mice. *E* and *F,* representative real time PCR analysis. INS-1 cells were treated with 100 mm ethanol in the presence or absence of 100 μm 4-methylpyrazole (*E*) or l-NMMA (*NMMA*) (100 μm), uric acid (*UA*) (100 μm), or deferoxamine (*DFO*) (50 μm) (*F*) and subjected to real time-PCR analysis (*, *p* < 0.01; **, *p* < 0.05). *Sal*, saline. *G* and *H, Gck* promoter activity. Twenty four hours after transfection, INS-1 cells were treated with 50 or 100 mm ethanol for 24 h in the presence or absence of 4-methypyrazole (*G*) and l-NMMA, uric acid, or deferoxamine (*H*), and then luciferase activity was measured (*, *p* < 0.01; **, *p* < 0.05). The data were normalized to β-galactosidase activity. All data are representative of three independent experiments. *CTL*, control.

In agreement with previous reports (11), *Gck* expression was reduced in the liver and pancreas of ethanol-fed mice and in ethanol-exposed pancreatic β-cells, and this reduction was correlated with increased hepatic steatosis and reduced islet cell mass (Fig. 1, *A–C*). Similarly, mRNA levels (Fig. 1, *D–F*) of *Gck* and *Glut2*, a glucose transporter, and the Luc activity (Fig. 1, *G* and *H*) of rat pancreatic *Gck* promoter pRGP-1003/Luc were significantly reduced in islet cells or INS-1 cells exposed to 100 mm ethanol, which was ameliorated by 4-methylpyrazole, an inhibitor of cytochrome P4502E1 (32), or peroxynitrite scavengers such as l-NMMA, uric acid, and deferoxamine (11). These results suggest that ethanol metabolism and peroxynitrites play a critical role in ethanol-mediated reduction of *Gck* transcriptional activity.

**FIGURE 2.**
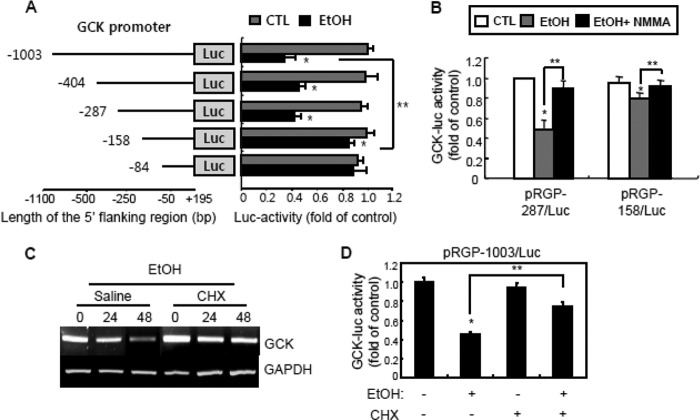
***De novo* protein synthesis is required for ethanol-reduced *Gck* expression.** *A,* INS-1 cells were transfected with constructs containing various lengths of serial deletions of the 5′-flanking region on the rat glucokinase promoter and then treated with 100 mm ethanol. After treatment for 24 h, *Gck* promoter activities were measured (*, *p* < 0.01; **, *p* < 0.05). *B, Gck*-Luc activity. Cells transfected with pRGP-287/Luc or pRGP-158/Luc construct were treated with ethanol and/or l-NMMA (*NMMA*) (*, *p* < 0.01; **, *p* < 0.05). *CTL*, control. *C,* stability of *Gck* mRNA. INS-1 cells were pretreated with cycloheximide (*CHX*) (10 μm) and then treated with ethanol. The samples were subjected to RT-PCR. *D,* cycloheximide inhibits ethanol-reduced *Gck* promoter activity (*, *p* < 0.01; **, *p* < 0.05). All data are representative of three independent experiments.

To determine the region of *Gck* promoter crucial for ethanol-mediated *Gck* down-regulation, we overexpressed rat *Gck* promoter constructs with 5′-serial deletions and analyzed their responsiveness to ethanol treatment in INS-1 cells (Fig. 2*A*). Ethanol treatment significantly decreased the Luc activity of the *Gck* promoter between −1003 and −287 bp, although we did not detect significant reductions between −158 and −84 bp, suggesting that the consensus binding site for a repressive transcription factor induced by ethanol was located between −287 and −158 bp. These results were confirmed using the pRGP-287/Luc and pRGP-158/Luc constructs (Fig. 2*B*). Also, we know that *de novo* protein synthesis as a repressive transcriptional factor is required for ethanol-repressed *Gck* transcriptional activity using cycloheximide, a *de novo* protein synthesis inhibitor (Fig. 2, *C* and *D*).

##### Ethanol-enhanced Atf3 Plays a Critical Role in the Down-regulation of Gck Transcriptional Activity

**FIGURE 3.**
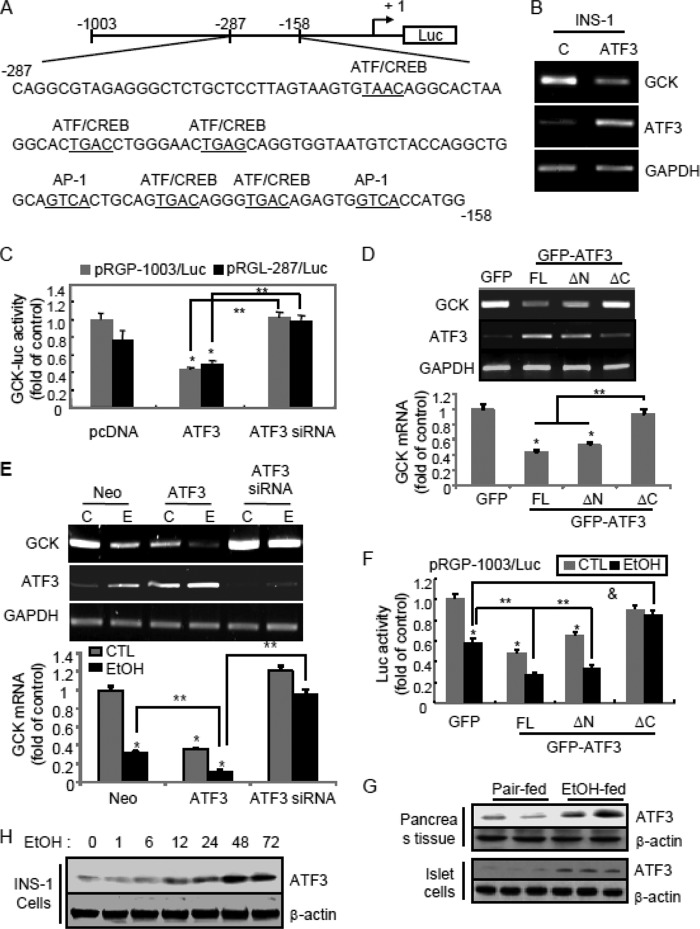
**Ethanol-mediated down-regulation of *Gck* gene expression depends on Atf3 induction.** *A,* schematic representation of rat *Gck* promoter (−287/−158). The putative binding sites for Atf/Creb and AP-1 are *underlined*. Their putative binding sites were searched using the TRANSFAC transcription factor binding database. *B,* RT-PCR analysis after transfection with *ATF3* cDNA into the INS-1 cells. *CTL*, empty vector-transfected cells. *C,* Atf3 inhibits *Gck* promoter activity. INS-1 cells were cotransfected with pRGP-1003/Luc or pRGL-287/Luc vectors and Atf3 or *Atf3* siRNA (*, *p* < 0.01; **, *p* < 0.05). *D,* RT-PCR analysis in the full-length (*FL*) *ATF3*, N-terminal domain-deleted (Δ*N*) or C-terminal domain-deleted *ATF3* (Δ*C*)-transfected cells (*, *p* < 0.01; **, *p* < 0.05). *E,* RT-PCR analysis. INS-1 cells were transfected with *ATF3* cDNA or *Atf3* siRNA and then exposed to 100 mm ethanol (*E*) (*, *p* < 0.01; **, *p* < 0.05). *F, Gck*-luciferase activity. Atf3 potentiates ethanol-reduced pRGP-1003/Luc activity (*, *p* < 0.01; **, *p* < 0.05; &, *p* < 0.005). *G* and *H,* Atf3 expression in pancreas tissues and islet cells isolated from ethanol-fed mice (*G*) or ethanol-treated INS-1 cells (*H*). The results represent the average ± S.E. from three independent experiments. *CTL,* control.

To understand the molecular mechanisms by which ethanol inhibits *Gck* promoter activity, we investigated how *Gck* promoter activity is regulated by searching a 129-bp consensus sequence between −287 and −158 bp of the 5′-flanking region of the *Gck* promoter using the TRANSFAC transcription factor binding database. Illustrated in Fig. 3*A*, five potential binding sites for Atf/Creb that contained the TAAC, TGAA, TGAG, or TGAC element, in addition to two binding sites for Ap-1 (GTCA), were found in the *Gck* promoter region (Fig. 3*A*). Because stress-inducible Atf3 is still considered as a dual-face transcription factor that activates or represses a gene expression (13), we first determined the role of Atf3 in repressing *Gck* transcriptional activity. *Gck* expression and the pRGP-1003/Luc or pRGP-287/Luc promoter activities were decreased by Atf3 overexpression but not by *Atf3* siRNA or C-terminal domain-deleted *ATF3*(ΔC) (Fig. 3, *B–F*). So, we wondered whether ethanol exposure affects *Atf3* gene expression *in vivo* and *in vitro* (Fig. 3, *G* and *H*). Atf3 was strongly increased in pancreatic tissue and islet cells of ethanol-fed mice or ethanol-treated INS-1 cells, indicating that *de novo* protein synthesis of Atf3 protein by ethanol may act as a negative transcription regulator to reduce *Gck* gene expression.

##### Atf3 Directly Binds to the Putative Atf/Creb Site on the Gck Promoter

**FIGURE 4.**
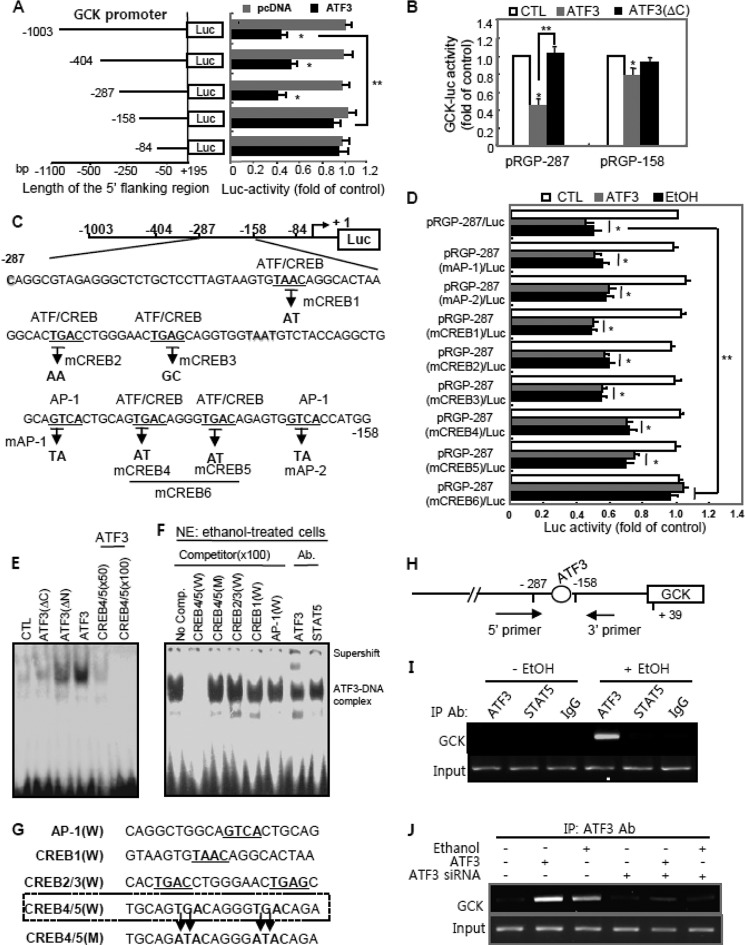
**Atf3 directly binds to the putative Atf/Creb site in the *Gck* promoter.** *A,* serially deleted constructs of *Gck* promoter were cotransfected with *ATF3* and measured luciferase activity (*, *p* < 0.01; **, *p* < 0.05). *B, Gck*-Luc activities were measured in cells cotransfected with pRGP-287 or pRGP-158 and *ATF3* or *ATF3*(Δ*C*) (*, *p* < 0.01; **, *p* < 0.05). *C,* point-mutated sequences of putative Atf3-binding sites in the *Gck* promoter. *D,* effects of Atf3 or ethanol on the activities of point-mutated pRGP-287/Luc promoter constructs (*, *p* < 0.01; **, *p* < 0.05). *E* and *F,* after transfection or ethanol treatment, EMSA was performed using *Creb4/5*(W) oligonucleotide. *NE*, nuclear extracts; *Ab*, antibody. *G,* oligonucleotides for EMSA and competition experiments. *H,* diagram showing the region between −283 and −135 of *Gck* promoter in the ChIP assay. *I* and *J,* ChIP assays. After transfection or treatment, immunoprecipitated (*IP*) DNA was amplified by PCR using the specific primers covering the Atf/Creb-binding sites (from −283 to −135). The results represent the average ± S.E. from three independent experiments. *CTL,* control.

Fig. 4*A* shows that ATF3 overexpression repressed *Gck* transcriptional activity through a direct interaction with the consensus binding sites of Atf/Creb located between −287 and −158 bp of the *Gck* promoter. The specific role of Atf3 within the proximal −287-bp region of the *Gck* promoter was confirmed by cotransfection with *ATF3* or inactive *ATF3*(ΔC) and pRGP-287/Luc or pRGP-158/Luc (Fig. 4*B*). The pRGP-287/Luc activity was significantly decreased by *Atf3*, but there was a small reduction by *Atf3* in pRGP-158/Luc-transfected cells. A TRANSFAC search revealed that the pRGP-158/Luc promoter also has two potential binding sites for Atf/Creb (TGAC) and one for Ap-1 (GTCA), but mutations of their consensus elements were not responsive to *Atf3* (data not shown). Therefore, to confirm the functional activity of putative binding sites for *Atf3* in pRGP-287/Luc, plasmids containing the putative binding sites were reconstructed by point mutagenesis (Fig. 4*C*). As shown in Fig. 4*D*, only pRGP-287(*mCreb6*)/Luc, which contains point mutations at the neighboring sites for *mCreb4* and *mCreb5*, did not respond to *ATF3* overexpression or ethanol treatment, whereas other constructs containing potential Atf3-binding site mutations still responded to them, suggesting that *Creb4* and *Creb5*, located between −185 and −174 bp of the *Gck* promoter, are essential for the inhibitory responsiveness of ethanol-induced Atf3. These are also confirmed by electrophoretic mobility shift assays (EMSAs) using a labeled oligonucleotide covering the −190 to −171-bp region (Creb4/5(W)) in cells overexpressing *ATF3*, *ATF3*(ΔC), or *ATF3*(ΔN) (Fig. 4*E*). Binding complexes were observed in nuclear extracts of *ATF3*- or *ATF3*(ΔN)-transfected cells but not in those of *ATF3*(ΔC)-transfected cells. Addition of an excess amount of the unlabeled competitor *Creb4/5*(W) (50- or 100-fold) completely blocked the binding of *Atf3* to the *Gck* promoter. To increase the specificity of the −190 to −171-bp region, three 20-bp wild-type oligonucleotides (*Ap-1*(W), *Creb1*(W), and *Creb2/3*(W)) that cover the putative binding site containing the sequence of response elements for Ap-1 or Atf/Crebs, respectively, and the mutated oligonucleotide *Creb4/5*(M), originating from wild-type *Creb4/5*(W), were designed (Fig. 4*G*). A 100-fold molar excess of unlabeled oligonucleotide for wild-type *Creb4/5*(W) (*2nd lane*) completely abolished the formation of the binding complex in nuclear extracts of ethanol-treated cells, whereas any oligonucleotides for mutated *Creb4/5*(M), including wild-type *Ap-1*(W), *Creb1*(W), and *Creb2/3*(W), did not compete (Fig. 4*F*). The specificity of Atf3 in the binding complexes was also confirmed in the results of a supershift assay using an anti-Atf3 antibody. The Atf3-DNA binding was much stronger in ethanol-treated INS-1 cells (no competitor lane) than those in nontreated cells (control). The recruitment of Atf3 to the *Gck* promoter was confirmed *in vivo* by chromatin immunoprecipitation. A 149-bp PCR product covering the putative Atf/Creb (from −283 to −135) of *Gck* promoter was detected in the nuclear extracts of ethanol-treated cells, but not in untreated cells, immunoprecipitated with the anti-Atf3 antibody (Fig. 4, *H* and *I*). There was no binding in chromatin immunoprecipitated with the anti-Stat5 antibody. As well, chromatin binding in anti-Atf3 immunoprecipitates was significantly increased in Atf3-transfected or ethanol-treated cells, which was strongly decreased by *Atf3* siRNA (Fig. 4*J*), indicating that Atf3 is specifically recruited to Atf/Creb binding region of the Gck promoter to result in a decrease of its transcriptional activity in ethanol-treated cells.

##### Ethanol and Atf3 Counteract the Positive Effect of Pdx-1 on Gck Transcriptional Regulation

**FIGURE 5.**
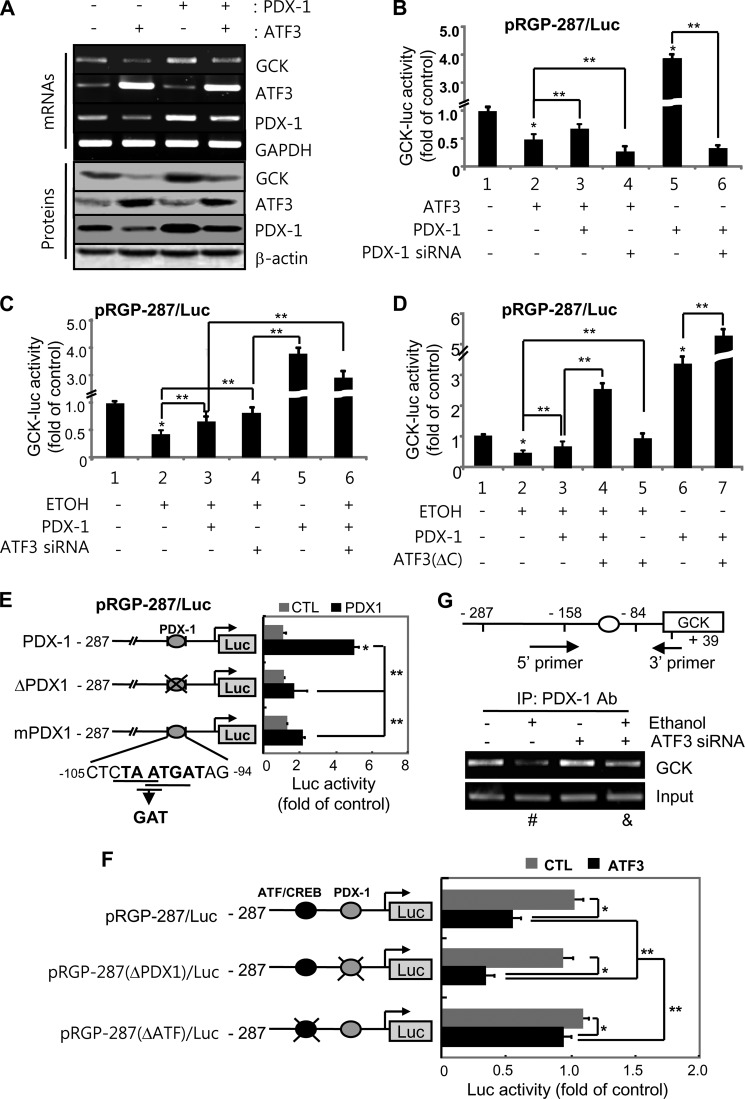
**Atf3 counteracts Pdx-1-mediated *Gck* transcriptional activity.** *A,* INS-1 cells were transfected with *Pdx-1* and/or *ATF3* and subjected to the semiquantitative RT-PCR (*upper panel*) and Western blotting (*bottom panel*). *B–D,* Pdx-1 restored Atf3 or ethanol-reduced pRGP-287/Luc activity. Twenty four hours after transfection with *Pdx-1* siRNA (*B*), *Atf3* siRNA (*C*), or *ATF3*(ΔC) (*D*), the luciferase activity was measured (*, *p* < 0.01; **, *p* < 0.05). *E,* identification of Pdx-1-binding sites on pRGP-287/Luc promoter. Deletion- or point-mutated constructs of putative Pdx-1-binding sites within −105 and −94 region of pRGP-287/Luc vector were generated and transfected into the cells (*, *p* < 0.01; **, *p* < 0.05). *F, ATF3* cDNA was cotransfected into the cells together with pRGP-287(Δ*Pdx-1*)/Luc or pRGP-287(Δ*Atf*)/Luc vector and measured the luciferase activity (*, *p* < 0.01; **, *p* < 0.05). *G,* ChIP assay for Pdx-1 binding to the *Gck* promoter. The immunoprecipitated (*IP*) DNAs were used as templates for PCR with primers covering Pdx-1-binding sites from −127 to −28 bp. The results represent the average ± S.E. from three independent experiments. *Ab,* antibody; *CTL,* control.

Although the effects of Pdx-1 on *Gck* expression are controversial (33), our data show the functional significance of the inter-regulation between Atf3 and Pdx-1 in *Gck* expression (Fig. 5*A*). The reduction of the *Gck* promoter activity by Atf3 or ethanol was potently increased by *Pdx-1* depletion (Fig. 5*B*), whereas the recovering effects of Pdx-1 were potentiated by Atf3 silencing or *ATF3*(ΔC) (Fig. 5, *C* and *D*), suggesting that Pdx-1 may counteract the repressive effects of Atf3 or ethanol on *Gck* transcriptional activity. Two putative Pdx-1-binding sites, TAAT and TGAT from −105 to −94 bp, were 72 bp from the core Atf/Creb binding sequence in the pRGP-287/Luc reporter (Fig. 5*E*). To determine whether these sites include the binding region for Pdx-1, we generated constructs via the deletion (−105/−94 bp, Δ*Pdx-1*) or point mutation (ATG→GAT, mPdx-1) of putative binding sites for Pdx-1 in the pRGP-287/Luc-promoter. Pdx-1-enhanced pRGP-287/Luc activity was significantly decreased in pRGP-287(Δ*Pdx-1*)/Luc or pRGP-287(*mPdx-1*)/Luc-transfected cells, indicating that the conserved binding site of Pdx-1 is located between −105 and −94 bp of the *Gck* promoter. The counteracting roles of Atf3 and Pdx-1 on *Gck* transcriptional regulation were further confirmed by using the construct in which Atf3-binding sites in *Gck* promoter are deleted (−190/−171, Δ*Atf*; Fig. 5*F*). The reduction of its activity by Atf3 was potentiated by the deletion of the Pdx-1-binding site (Δ*Pdx-1*), which was rescued by the deletion of the Atf3-binding site, indicating that Atf3 and Pdx-1 may have opposite effects on *Gck* transcriptional activity through a direct interaction. Next, we hypothesized that the negative effect of ethanol on Pdx-1-mediated *Gck* transcriptional activation may be caused via the up-regulation of Atf3, which prevents Pdx-1 recruitment to its putative binding site in the *Gck* promoter. To this, cells exposed to ethanol in the presence of *Atf3* siRNA were assayed by ChIP analysis using primer sequences covering the Pdx-1-binding site (from −127 to −28). The binding of Pdx-1 to the putative binding region of the *Gck* promoter was reduced in ethanol-treated cells but not in *Atf3* siRNA (Fig. 5*G*) or *ATF3*(ΔC)-transfected cells (data not shown), suggesting that induced Atf3 may indirectly suppress *Gck* transcriptional activity by preventing Pdx-1 recruitment to its putative binding site.

##### Ethanol-induced Atf3 Interacts with Pdx-1 and Subsequently Promotes the Recruitment of Hdac1/2 and Histone H3 Deacetylation on Gck Promoter

**FIGURE 6.**
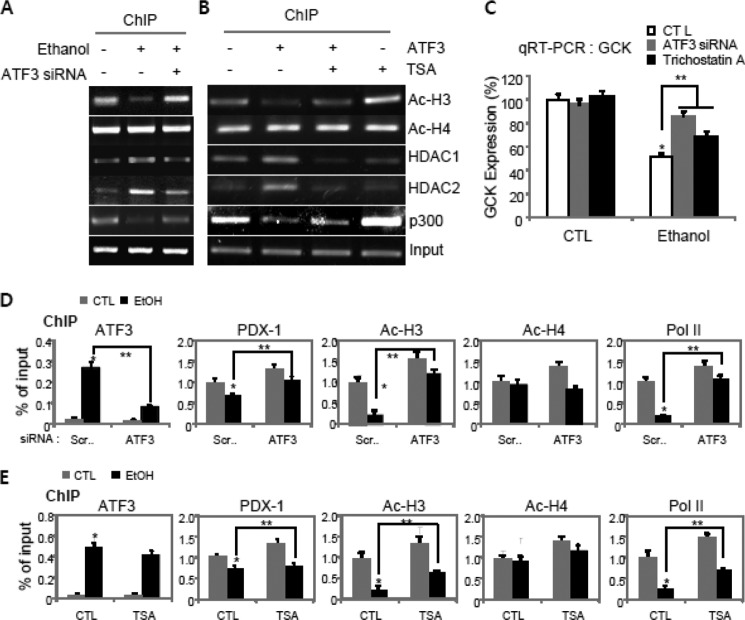
**Atf3 recruitment inhibits histone H3 acetylation and induces sequential recruitment of Hdac1/2.** *A* and *B,* ChIP assays were performed in ethanol-treated cells (*A*) and *ATF3*-transfected cells (*B*) exposed to *Atf3* siRNA or TSA (10 nm), respectively. The immunoprecipitated DNA was amplified by PCR using the specific primers covering the Pdx-1-binding sites. *C,* quantitative RT-PCR analysis for *Gck* expression (*, *p* < 0.01; **, *p* < 0.05). *D* and *E,* ChIP analyses for Atf3, Pdx-1, Ac-H3, Ac-H4, or polymerase II (*Pol II*) in cells treated with ethanol in the presence or absence of *Atf3* siRNA (*D*) or TSA (*E*). The immunoprecipitated chromatin was analyzed by quantitative PCR (*, *p* < 0.01; **, *p* < 0.05). The results represent the average ± S.E. from three independent experiments. *CTL,* control.

Next, to examine whether *Atf3* may inhibit Pdx-1-mediated *Gck* transcription by decreasing histone acetylation on the *Gck* promoter, we have quantified the amount of *Gck* gene promoter associated with acetylated histone H3 or H4. To this, ChIP analysis was performed by using the primers covering Pdx-1-binding sites from −127 to −28 bp. As expected, histone H3 acetylation on the *Gck* promoter was lower in ethanol-treated (Fig. 6*A*) or *ATF3*-overexpressed cells (Fig. 6*B*), correlated with reduction in p300 binding, whereas the recruitment of histone deacetylase, Hdac1 and -2, to the Pdx-1 binding region of *Gck* promoter was increased in ethanol-treated cells, which were markedly attenuated and reversely rescued by *Atf3* depletion or trichostatin A (TSA), an inhibitor of histone deacetylase (27). Concomitantly, *Gck* mRNA was significantly decreased by ethanol or *ATF3* overexpression, which was restored by TSA or *Atf3* siRNA (Fig. 6*C*). Also, quantitative PCR using ChIP samples revealed that ethanol-induced *Atf3* directly binds to the *Gck* promoter despite no Atf3-binding sites, whereas recruitment of Pdx-1, Ac-H3, and RNA polymerase II to the promoter was significantly reduced in ethanol-treated cells, which were reversely attenuated by *Atf3* depletion (Fig. 6*D*), demonstrating the specificity of their recruitment by Atf3. Additionally, the inhibition of histone deacetylase activity by TSA may partially restore the recruitment of Pdx-1, Ac-H3, and polymerase II to the *Gck* promoter after ethanol treatment, although there was no impact on Atf3 recruitment (Fig. 6*E*), indicating that Atf3 may be an upstream regulator of Hdac1/2 activation.

##### Atf3 Attenuates Pdx-1-dependent Gck Transcriptional Activity by Enhancing the Physical Interaction with Hdac1

**FIGURE 7.**
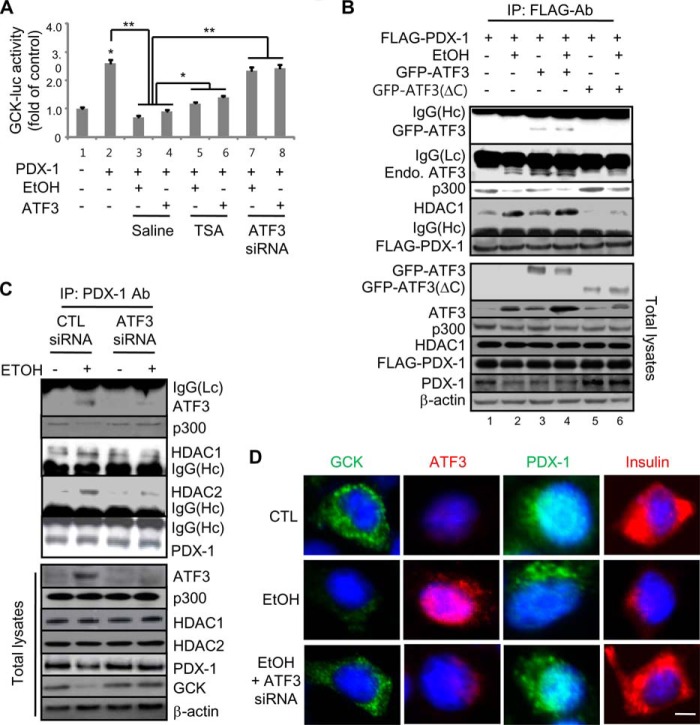
**Atf3 inhibits Gck transcriptional activity via physical interaction with HDAC1/Pdx-1.** *A, Gck*-Luc activity. Atf3 inhibits Pdx-1-mediated pRGP-158/Luc promoter activity, which is rescued by TSA or *Atf3* siRNA (*, *p* < 0.01; **, *p* < 0.05). *B,* interaction assay for *Flag-Pdx-1*. After ethanol treatment into the cells cotransfected with *Flag-Pdx-1*, *GFP-ATF3*, or *GFP-ATF3*(ΔC), the lysates were immunoprecipitated (*IP*) with FLAG antibody (*Ab*) and subjected to Western blotting. *C, in vivo* binding assay for endogenous Pdx-1. INS-1 cells were treated with 100 mm ethanol in the presence or absence of *Atf3* siRNA, and then we performed the interaction assay. *D,* immunocytochemistry analyses for Gck, Atf3, Pdx-1, and insulin were performed in the cells treated with ethanol and/or *Atf3* siRNA (*scale bars,* 100 μm). The results represent the average ± S.E. from three independent experiments.

We next investigated whether ethanol-induced Atf3 could inhibit Pdx-1-dependent *Gck* transcriptional activity by enhancing the activity of histone deacetylase (Fig. 7*A*). The increase of pRGP-158/Luc activity by Pdx-1 was significantly decreased by ethanol treatment or *ATF3* overexpression, which was restored by TSA or *Atf3* siRNA, indicating that the activated histone deacetylase may play an essential role in the inhibitory effects of Atf3 on Pdx-1-mediated *Gck* transcriptional activity. Also, ethanol-induced Atf3 inhibits Pdx-1-mediated *Gck* transcriptional activity by triggering a physical *in vitro* and *in vivo* interaction of Pdx-1 with Hdac1 rather than p300 (Fig. 7, *B* and *C*). Immunoprecipitated Pdx-1 directly interacted with endogenous Atf3 induced by ethanol or overexpressed *GFP-ATF3* but not with *GFP-ATF3*(ΔC). Conversely, the interaction of p300 with *Flag-Pdx-1* was significantly decreased by ethanol treatment or *ATF3* overexpression. Additionally, *Gck* down-regulation in ethanol-treated β-cells was correlated with the induction of Atf3 and the reduction of nuclear localization of Pdx-1, which were reversely rescued by Atf3 siRNA (Fig. 7*D*).

##### In Vivo Atf3 Silencing Ameliorates Metabolic Syndrome and β-Cell Damage in Ethanol-fed Mice

**FIGURE 8.**
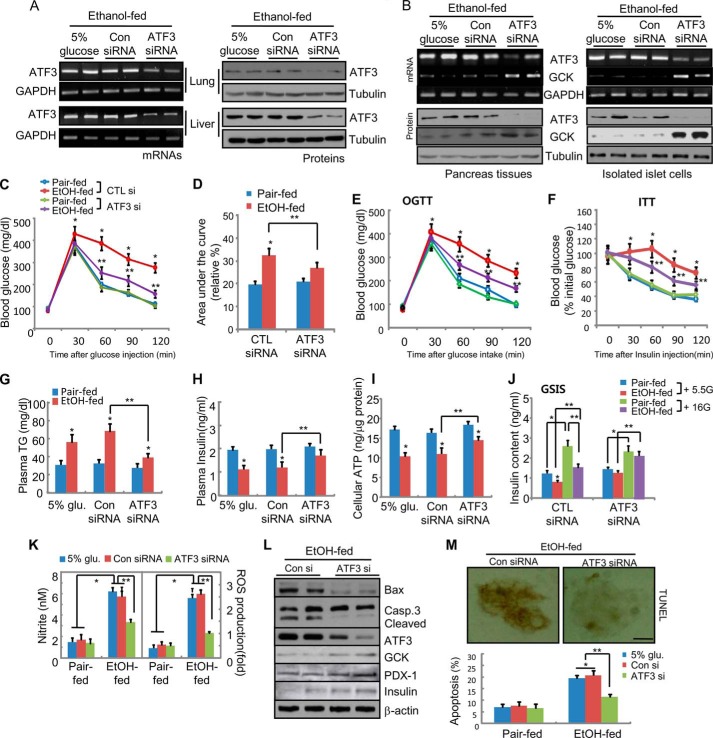
***In vivo Atf3* knock-out ameliorates the impairment of glucose metabolism and β-cell damage in ethanol-fed mice.** Male 6-week-old C57BL/6J mice (*n* = 8, each group) were fed an ethanol diet, and after 2 weeks, the mice were intravenously injected with *Atf3* siRNA six times with a 3-day interval between injections, then maintained for 14 weeks, and sacrificed. *A,* expression of *Atf3* mRNA and proteins by RT-PCR (*left panel*) and Western blotting (*right panel*) in the tissues of lung and liver of the sacrificed mice. *B, in vivo* silencing of *Atf3* induced the increase of *Gck* expression down-regulated in ethanol-fed mice. RT-PCR (*upper panel*) and Western blotting (*lower panel*) were performed in pancreas tissues (*left panel*) and isolated islet cells (*right panel*) of *Atf3* siRNA-injected mice. *C* and *D, Atf3* silencing improves glucose tolerance (*C*). Areas under the curve (*D*) are shown. *, *p* < 0.01; **, *p* < 0.05. *E,* oral glucose tolerance test (*OGTT*, 1g/kg; *, *p* < 0.01; **, *p* < 0.05). *F,* insulin tolerance (*ITT*; *, *p* < 0.01; **, *p* < 0.05). *G,* plasma triglyceride levels (*, *p* < 0.01; **, *p* < 0.05). *H,* plasma insulin levels (*, *p* < 0.01; **, *p* < 0.05). *I,* cellular ATP production in islet cells isolated from each group mice (*, *p* < 0.01; **, *p* < 0.05). *J,* glucose-stimulated insulin release was measured in islets exposed to 5.5 and 16 mm/liter glucose. *GSIS,* glucose-stimulated insulin secretion. *, *p* < 0.01; **, *p* < 0.05. *K,* NO and ROS production (*, *p* < 0.01; **, *p* < 0.05). *L,* apoptosis-related protein expression. *M,* TUNEL assay in isolated islet cells (*top panel*, ×100). *Casp.,* caspase. TUNEL-positive apoptotic cells were quantified (*bottom panel*, *, *p* < 0.01; **, *p* < 0.05). The results represent the average ± S.E. from three independent experiments.

To confirm the specific upstream role of Atf3 on ethanol consumption-induced *Gck* down-regulation and metabolic syndrome, a loss-of-function study was performed through *in vivo* Atf3 siRNA injection. Among four siRNA candidates, we selected 356-Atf3 siRNA, which had the highest efficiency for Atf3 silencing in various tissues (Fig. 8, *A* and *B*). As expected, the impaired glucose tolerance (*GTT* and OGTT; Fig. 8, *C–E*) and insulin tolerance (*ITT*; Fig. 8*F*) induced by ethanol consumption were markedly inhibited by the administration of Atf3 siRNA. Triglyceride accumulation and reduced plasma insulin levels in ethanol-fed mice were also attenuated by Atf3 knockdown (Fig. 8, *G* and *H*), correlated with restoring the reduced ATP (Fig. 8*I*). Similarly, the reduction in glucose-stimulated insulin secretion (*GSIS*; Fig. 8*J*) and the production of nitrite or ROS (Fig. 8*K*) in islet cells of ethanol-fed mice also depended on the presence of Atf3. Together with the amelioration of pancreatic β-cell dysfunction, *in vivo Atf3* silencing inhibited ethanol-induced β-cell apoptosis, which was associated with the restoration of *Gck*, insulin, and Pdx-1 expression (Fig. 8, *L* and *M*). These results suggest that the *Atf3* gene is associated with the induction of T2D and alcohol consumption-induced metabolic impairment and thus may play a major negative regulator for glucose homeostasis.

## DISCUSSION

**FIGURE 9.**
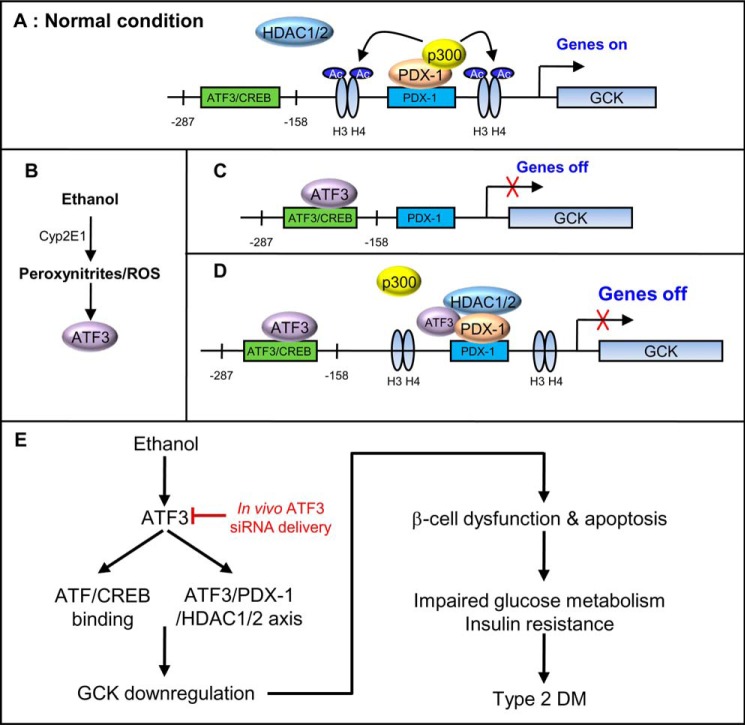
**Proposed model by which Atf3 induces *Gck* down-regulation and metabolic syndrome in chronic ethanol-fed mice.** *A,* in normal conditions Pdx-1 interacts with p300 and binds to the putative binding site of the *Gck* promoter, followed by the acetylation of histone H3 and H4, and then activates *Gck* transcription. *B,* ethanol induces Atf3 via the production of peroxynitrite and ROS, which depends on ethanol metabolism. *C,* ethanol-induced Atf3 directly binds to Atf/Creb-binding site of *Gck* promoter and then inhibits *Gck* transcription. *D,* as well as the direct binding of Atf3 to the Atf/Creb-binding site of the *Gck* promoter, ethanol-induced Atf3 also facilitates ethanol-reduced Gck transcriptional activity via the direct interaction with Pdx-1 and the recruitment of Hdac1/2 rather than p300. *E, in vivo Atf3* silencing ameliorates *Gck* down-regulation and metabolic syndrome induced by ethanol-induced Atf3 (*red*).

In this study, we provide evidence that ethanol-induced Atf3 suppresses *Gck* gene expression through direct interaction with the Atf/Creb-binding site (−185/−174) of the *Gck* promoter. Additionally, Atf3 directly interacts with Pdx-1 and specifically recruits Hdac1/2 to the *Gck* promoter in ethanol-treated β-cells, thereby repressing functional Pdx-1 on *Gck* transcriptional regulation. *In vivo Atf3* silencing ameliorates ethanol-mediated metabolic syndrome and pancreatic β-cell dysfunction. All of these findings are summarized in Fig. 9.

This study demonstrates a functional role of Atf3 as a major upstream regulator that negatively regulates *Gck* transcriptional activity in ethanol-fed mice, based on the following observations. (i) The reduction of *Gck* transcriptional activity by ethanol was potentiated by Atf3, which was inhibited by *Atf3* depletion. (ii) Two putative Atf3-binding sites that are essential for the inhibitory responsiveness by Atf3 were identified between −185 and −174 bp of the *Gck* promoter. (iii) Atf3 also binds to the Pdx-1-binding sites between −102 and −92 of the *Gck* promoter, resulting in chromatin remodeling via histone H3 deacetylation.

Several studies have suggested that Pdx-1 is a β-cell master gene that regulates the expression of β-cell-specific genes such as insulin, *Glut2*, and *Gck* (34–36). Although we previously suggested that activated Atf3 directly interacted with Pdx-1 (20), the correlation of Pdx-1 function with *Gck* expression in the transcriptional hierarchy is unclear, and the upstream regulator that serves as the activator or repressor for Pdx-1-mediated gene expression is also unknown. This study suggested the opposing effects of Atf3 on Pdx-1-mediated *Gck* transcriptional regulation, which were caused by their direct interaction *in vitro* and *in vivo* (Figs. 5–7). *Pdx-1* overexpression reversely attenuated or rescued the inhibitory effects of Atf3 on *Gck* expression, but how Pdx-1 counteracts Atf3-repressed *Gck* transcriptional activity in response to ethanol is still unclear. Our data show that two putative Pdx-1-binding sites may be essential for counteracting the inhibitory effects of Atf3 on *Gck* transcriptional activity by using a pRGP-287/Luc with a deleted or point-mutated Pdx-1-binding site (Fig. 5*D*). Despite the lack of an Atf3-binding site, Atf3 still induced a small reduction in pRGP-287(Δ*Atf*)/Luc activity, which was similar to the small reduction in the pRGP-158/Luc promoter (Fig. 4), suggesting that Atf3 may also inhibit Pdx-1 functional activity within the −158-bp region. The association of Pdx-1 with insulin promoter is enhanced by its interaction with p300, and the initial acetylation of histone H4 may stabilize Pdx-1 binding (37). However, when glucose levels are low, Pdx-1 interacts with Hdac1/2 rather than p300, and the complexes are recruited to the insulin promoter, thus leading to histone H4 deacetylation and insulin gene down-regulation (38). Similarly, ethanol-induced Atf3 may first interact with Pdx-1 and subsequently prevent the formation of Pdx-1/p300, followed by chromatin remodeling via alternative recruitment of histone deacetylase to the consensus Pdx-1-binding sites of the *Gck* promoter. Furthermore, because the primers used in ChIP assay cover the putative Pdx-1-binding sites (−28/−127) on the pRGP-158/Luc promoter, the product does not contain Atf3-binding sites and may present only low responsiveness for Atf3. Therefore, it is important to determine how Atf3 acts as a negative upstream regulator for Pdx-1 functional activity at the −158-bp proximal region of the *Gck* promoter. Quantitative ChIP assay revealed that ethanol-induced Atf3 binds to the proximal −158-bp region, whereas recruitments of Pdx-1, acetylated H3, and RNA polymerase II were significantly decreased in ethanol-treated cells, which were ameliorated by Atf3 depletion (Fig. 6*D*), demonstrating that Atf3 association with *Gck* promoter is a critical step for histone H3 deacetylation. Despite the decreased recruitment to the *Gck* promoter and expression of Pdx-1 in ethanol-treated cells, how the formation of Atf3/Pdx-1/Hdac1/2 complexes is increased in response to ethanol is unknown. Previously, it was demonstrated that Pdx-1 phosphorylation may determine the interaction of Pdx-1 with p300/Cbp or Hdac1/2 in the regulation of the insulin gene, which may depend on the concentration of glucose (35–37). Similarly, it is possible that ethanol-induced Atf3 directly interacts with Pdx-1, subsequently decreasing Pdx-1 phosphorylation and recruiting Hdac1/2 rather than p300 (Fig. 7); this possibility should be explored further.

We also confirmed the specific upstream role of Atf3 in chronic ethanol-induced *Gck* down-regulation and pancreatic β-cell dysfunction by performing a loss-of-function study using the *in vivo Atf3* siRNA delivery system (Fig. 8). *In vivo Atf3* silencing ameliorated the impaired glucose metabolism and pancreatic β-cell dysfunction in ethanol-treated cells, and therefore prevented the development of T2D by reducing RNS production. However, in our model, the injected *Atf3* siRNA may act on several organs such as liver and lung and is not specific to pancreatic β-cells (Fig. 8, *A* and *B*). Therefore, to define the precise role of Atf3 in ethanol-mediated *Gck* down-regulation and β-cell dysfunction, additional studies using pancreas-specific knock-out models are required. Nevertheless, we propose that *in vivo Atf3* silencing may be sufficient to improve metabolic syndrome and pancreatic β-cell dysfunction by ameliorating ethanol-induced *Gck* down-regulation. In contrast with the ameliorating effects of *Atf3*-silencing mice *in vivo*, it will be possible that *Atf3* knock-out mice may conversely potentiate ethanol-mediated *Gck* down-regulation and β-cell dysfunction. Because stress-inducible *Atf3* is a dual-face transcription factor that activates or represses gene expression (13), it may initially develop a compensatory or adaptive response for the acute oxidative stress or endoplasmic reticulum stress. Although the data are not shown here, we have determined genetic susceptibility of Atf3 to T2D risk by performing conditional analyses using our previous GWAS data (KARE study) based on the Korean population (39, 40). Furthermore, we have identified a noncoding variant in the *Atf3* gene as a novel predisposing factor to T2D after controlling for alcohol consumption in T2D groups (data not shown). From these results, we propose that Atf3 may play an important role in ethanol-mediated metabolic alteration, and it may be strongly associated with T2D.

In this study, the direct interaction of Atf3 with the Atf/Creb-binding site of the *Gck* promoter may have been a major molecular mechanism by which ethanol reduced *Gck* expression and pancreatic β-cell dysfunction and apoptosis. Also, Atf3-dependent interaction of Pdx-1 with Hdac1/2 and subsequent inactivation of Pdx-1 and chromatin remodeling may represent an additional molecular mechanism by which ethanol-induced Atf3 decreases *Gck* down-regulation (Fig. 9). Taken together, these results show for the first time that Atf3 may act as an upstream negative regulator for *Gck* transcriptional activation via direct binding to the putative binding site and by indirect binding with Pdx-1/Hdac1/2 on the *Gck* promoter. Additionally, our findings suggest that strategies based on the inhibition of Atf3 may be of benefit in the treatment of ethanol-mediated glucose impairment and metabolic syndrome.

## Footnotes

2 The abbreviations used are:

T2D: type 2 diabetes
Gck: glucokinase (hexokinase IV)
ROS: reactive oxygen species
Hdac: histone deacetylase
Creb: cAMP-response element-binding protein
Luc: luciferase
TSA: trichostatin A.
